# CDK6-Dependent, CDK4-Independent Synovial Hyperplasia in Arthritic Mice and Tumor Necrosis Factor-α-Induced Proliferation of Synovial Fibroblasts

**DOI:** 10.3390/ijms26031151

**Published:** 2025-01-28

**Authors:** Rie Komatsu, Ryoji Fujii, Toru Ogasawara, Yuki Suzuki-Takahashi, Sandy Chen, Yodo Sugishita, Hisateru Niki, Kazuo Yudoh

**Affiliations:** 1 Institute of Medical Science, St. Marianna University School of Medicine, Kawasaki 216-8512, Kanagawa, Japan; komatsur@marianna-u.ac.jp (R.K.); y2takahashi@marianna-u.ac.jp (Y.S.-T.); chen.sandy@marianna-u.ac.jp (S.C.); yodo@marianna-u.ac.jp (Y.S.); yudo@marianna-u.ac.jp (K.Y.); 2 Department of Oral and Maxillofacial Surgery, Graduate School of Medicine, The University of Tokyo, Hongo, Bunkyo-ku, Tokyo 113-8655, Japan; togasawara-tky@umin.ac.jp; 3 Department of Orthopedic Surgery, St. Marianna University School of Medicine, Kawasaki 216-8511, Kanagawa, Japan; h2niki@marianna-u.ac.jp

**Keywords:** CDK4, CDK6, G1 phase progression, collagen-induced arthritis, synovial hyperplasia

## Abstract

Palbociclib, a dual CDK4/6 kinase inhibitor used for breast cancer, has been explored as a treatment option for rheumatoid arthritis (RA). Preclinical studies have reported palbociclib-induced myelosuppression, but no such effects have been observed in *Cdk4* or *Cdk6* single-deficient mice. Synoviocyte proliferation-associated in collagen-induced arthritis 1/serum amyloid A-like 1 (SPACIA1/SAAL1) is involved in G1 phase progression. Given that SPACIA1/SAAL1 upregulates *CDK6* (but not *CDK4*) expression, we aimed to determine whether suppressing CDK6 expression alone could prevent synovial hyperplasia without myelosuppression. The effects of CDK6 expression on TNF-α-induced rheumatoid arthritis synovial fibroblast (RASF) proliferation and synovial hyperplasia in collagen-induced arthritis (CIA) mice were investigated by modulating the transcriptional level with a *CDK6* expression inhibitor (indole-3-carbinol), *CDK6* small interfering RNA (siRNA), and *Cdk6*-deficient mice. Indole-3-carbinol or *CDK6* siRNA inhibited TNF-α-induced RASF proliferation without suppressing CDK4 expression and reduced retinoblastoma protein phosphorylation. In CIA mice, indole-3-carbinol did not cause myelosuppression, considerably delayed CIA onset and progression, and reduced arthritis severity. *Cdk6*-deficient mice showed similar improvements in CIA pathogenesis but had lower serum anti-type II collagen IgG levels. Notably, synovial hyperplasia was not observed in *Cdk6*-deficient mice. CIA-synovial hyperplasia depends on CDK6, but not CDK4, expression.

## 1. Introduction

Rheumatoid arthritis (RA) is a chronic autoimmune disease characterized by inflammation and hyperplasia of synovial membranes, accompanied by immune abnormalities [1]. Cytokine-stimulated rheumatoid arthritis synovial fibroblasts (RASFs) contribute to inflammation by secreting proteases and enzymes that initiate and sustain synovitis, making them promising targets for therapy [2,3]. Currently, methotrexate and biologics are the standard treatments for RA, with reportedly high efficacy [4]. However, approximately one-third of patients with RA do not adequately respond to current treatments, and maintaining remission is challenging [5,6,7].

We previously identified synoviocyte proliferation-associated in collagen-induced arthritis (CIA) 1/serum amyloid A-like 1 (SPACIA1/SAAL1) as a novel molecular target for RASF proliferation [8]. SPACIA1/SAAL1 is strongly expressed in RA synovial tissues but not in normal tissues and is an aggravating factor in the progression of collagen-induced arthritis in mouse models of RA. Furthermore, SPACIA1/SAAL1 plays a role in the G1 phase progression of RASF proliferation and the post-transcriptional regulation of *CDK6* mRNA stability in vitro [9].

CDK4 and CDK6 (CDK4/6) are serine–threonine kinases that form complexes with D-type cyclins to phosphorylate retinoblastoma protein (RB) and activate the E2F transcription factor to drive cell cycle progression from the G1 to S phase [10]. *Cdk4* or *Cdk6* single-deficient mice are viable and *Cdk4/6* double-deficient mice are not viable because of defective erythroid progenitor cell proliferation during late embryogenesis, indicating functional redundancy [11]. However, some non-redundant functions have been reported in *Cdk4* or *Cdk6* single-deficient mice [12,13].

Targeting CDK4/6 activity is a key strategy for cancer treatment. CDK4/6 inhibitors, such as palbociclib, abemaciclib, and ribociclib, are Food and Drug Administration (FDA)-approved drugs for breast cancer [14]. *CDK4* and *CDK6* have been identified as disease-susceptibility genes in RA [15,16]. Sekine et al. reported that CDK4/6 inhibitors ameliorated arthritis progression in CIA mice, indicating that inhibiting RASF proliferation could be a beneficial treatment for RA [17,18]. However, myelosuppression, including neutropenia, was commonly observed in mice treated with palbociclib [19]. Neutropenia caused by palbociclib has also been reported in patients with breast cancer, prompting dose reduction or treatment discontinuation [20]. The primary structures at the ATP binding region of CDK4/6 in which CDK4/6 inhibitors act competitively share 94% homology [21]. The inhibitors are highly selective for CDK4/6, but not for other cyclin-dependent kinases (CDKs); however, they cannot preferentially differentiate CDK6 [22].

As previously mentioned, SPACIA1/SAAL1 is associated with CDK6 expression in RASFs but has no effect on CDK4 expression. Functional redundancy of CDK4/6 depends on cell type and growth stimuli. While TNF-α stimulation increases the expression of CDK4/6 in breast cancer cells (MCF7), only CDK6 expression is upregulated in RASFs [23,24]. Whether the proliferative role of CDK6 in RASFs overlaps with that of CDK4 has not been investigated. We discovered distinct roles of CDK4/6 in the cell cycle progression of TNF-α-induced RASFs. We investigated how CDK6 inhibition with indole-3-carbinol (I3C) or systemic *Cdk6*-deficiency in a murine model of arthritis ameliorates the progression of pathological changes and suppresses synovial hyperplasia without affecting myelosuppression, particularly neutropenia.

## 2. Results

### 2.1. I3C, an Inhibitor of CDK6 Expression, Modulates Synovial Hyperplasia in CIA Mice

**Figure 1:**
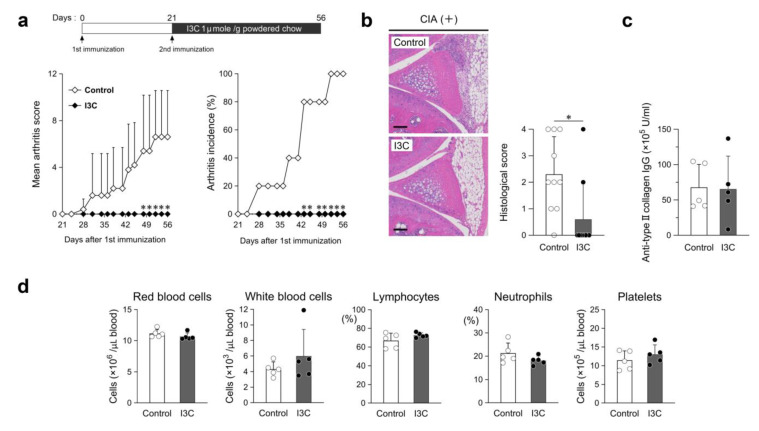
Anti-arthritic effect of indole-3-carbinol (I3C) in RA model mice. (**a**) Schematic representation of the I3C-mixed diet. Mean arthritis scores and incidence in control or I3C-administered (I3C) mice. (**b**) Images of HE-stained sagittal cross-section; scale bar = 100 μm. Histological scores of knee joints. (**c**) Serum anti-type II collagen IgG. (**d**) Peripheral blood cell counts indicating red blood cell, white blood cell, and platelet counts. Neutrophils and lymphocytes were classified by leucocyte morphology (%). Data in (**a**,**c**,**d**): n = 5 per group, (**b**): n = 10 per group. Closed or open circles represent individual data. Values are mean ± SD; * *p* < 0.05 versus control mice.

The anti-arthritic effect of I3C, an inhibitor known to suppress *CDK6* mRNA expression [25], in CIA mice was evaluated. An I3C-mixed diet was initiated immediately after the second immunization. In comparison with the control, I3C administration (1 μmole/g powdered chow) completely prevented swollen joints, and arthritis did not occur until the last day of observation (Figure 1a). The final knee histological score in the I3C-administered group was approximately 70% lower than that in the control group (mean, 2.3) (Figure 1b). Synovial hyperplasia (histological score, 3) and pannus formation (histological score, 4), which can cause irreversible bone destruction, were observed in 1 of 10 cases in the I3C-administered group across both knee tissues from five mice per group and in 4 of 10 cases in the control group. The serum anti-type II collagen IgG (anti-CII IgG) level remained unchanged between the groups (Figure 1c). The peripheral red blood cell, white blood cell, lymphocyte, neutrophil, and platelet counts were not noticeably different among the mice (Figure 1d).

### 2.2. Effects of I3C in Cultured RASFs

**Figure 2:**
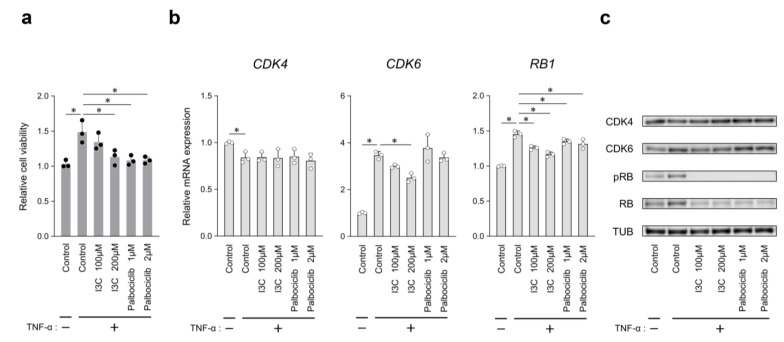
Effect of indole-3-carbinol (I3C) or palbociclib on G1 phase progression of TNF-α-induced RASF proliferation. (**a**) Relative cell viability after 72 h treatment with DMSO (Control), I3C (100 μM, 200 μM), and palbociclib (1 μM, 2 μM) in the presence of PBS or TNF-α (10 ng/mL). (**b**) *CDK4*, *CDK6*, and *RB1* mRNA expression levels after 6 h treatment with each inhibitor were normalized to *GAPDH* mRNA level. (**c**) Western blotting of cell lysates obtained after 24 h treatment with each inhibitor. Representative results from 3 independent experiments are shown here. Closed or open circles represent individual data. Values are mean ± SD of three biological replicates; * *p* < 0.05 versus TNF-α-stimulated RASF with DMSO (Control).

We evaluated the effect of I3C on TNF-α-induced RASF proliferation and *CDK4*, *CDK6*, and rb transcriptional corepressor 1 (*RB1*) mRNA and protein expression levels to confirm the mechanism by which I3C suppresses synovial hyperplasia, compared with the effects of palbociclib, a CDK4/6 kinases inhibitor. The number of viable cells increased by TNF-α stimulation was substantially reduced when 200 μM I3C was added. The growth-inhibitory effect of 200 μM I3C was similar to that of 1 μM palbociclib (Figure 2a). *CDK6* mRNA expression upregulated by TNF-α, decreased following the addition of 200 µM I3C. However, no marked change was observed following the addition of palbociclib, and similar results were observed for the CDK6 protein level (Figure 2b,c). *CDK4* mRNA expression was downregulated by TNF-α, but no apparent changes were observed at the mRNA and protein levels with either inhibitor (Figure 2b,c). TNF-α-induced *RB1* mRNA, a gene encoding RB (a target protein of CDK4/6), expression was slightly but statistically reduced by both inhibitors; however, both RB protein and phosphorylated RB (pRB) expression were almost completely prevented (Figure 2b,c).

### 2.3. Deletion of Cdk6 Suppressed Synovial Hyperplasia in CIA Mice

**Figure 3:**
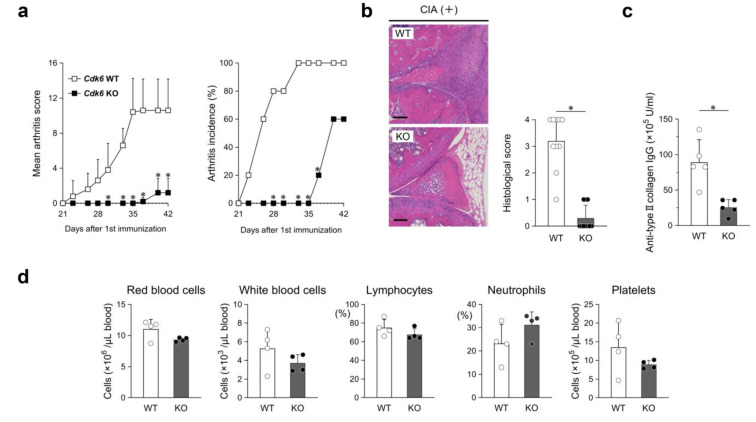
Contribution of CDK6 to pathogenesis in RA model mice. (**a**) Mean arthritis scores and incidence in wild-type (WT) and *Cdk6*-deficient (KO) mice. (**b**) Images of HE-stained sagittal cross-section; scale bar = 100 μm. Histological scores of knee joints. (**c**) Serum anti-type II collagen IgG. (**d**) Peripheral blood cell counts. Data in (**a**,**c**), n = 5 per group; (**b**), n = 10 per group; (**d**), n = 4 per group. Closed or open circles represent individual data. Values are mean ± SD; * *p* < 0.05 versus WT mice.

Given that the effect of I3C on *CDK6* mRNA expression in TNF-α-induced RASFs was moderate, CIA was induced in *Cdk6*-deficient (KO) mice to assess the role of CDK6 in synovial hyperplasia. The arthritis score of KO mice was lower than that of wild-type (WT) littermates, with marked differences observed from day 30 after the first immunization to the last day of observation (Figure 3a). The incidence of arthritis was also substantially delayed in KO mice, with no onset observed until day 35 (Figure 3a). The final knee histological score of KO mice was approximately 90% lower than that of WT mice (mean, 3.2) (Figure 3b). Synovial hyperplasia was not observed in any KO mice. In contrast to that in the I3C-administered group (Figure 1c), the serum anti-CII IgG level in KO mice decreased by approximately 70%, compared with that in WT mice (mean, 89 × 10^5^ U/mL) (Figure 3c). No obvious variation was detected in any of the peripheral blood parameters (Figure 3d).

### 2.4. CDK6, but Not CDK4, Is Selectively Involved in TNF-α-Induced RASF Proliferation

**Figure 4:**
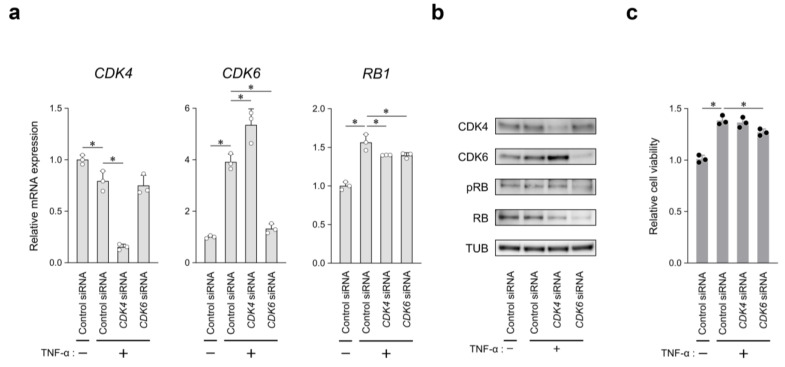
Contribution of CDK4/6 to TNF-α-induced RASF proliferation. (**a**) *CDK4*, *CDK6*, and *RB1* mRNA expression levels upon transfection of *CDK4* or *CDK6* siRNA. After 24 h siRNA transfection, cells were cultured in DMEM containing PBS or TNF-α (10 ng/mL) for 6 h and normalized to *GAPDH* level. (**b**) Western blotting of cell lysates transfected with siRNA and then treated for 24 h with PBS or TNF-α. Representative results from 3 independent experiments are shown here. (**c**) Relative viability of RASFs transfected with siRNA and treated for 72 h with PBS or TNF-α. Closed or open circles represent individual data. Values are mean ± SD of three biological replicates; * *p* < 0.05 versus TNF-α-stimulated RASF transfected with control siRNA.

To determine the contribution of TNF-α-induced CDK6, but not CDK4 in RASFs, we performed cell proliferation assays and analyzed the pRB level under siRNA suppression of each gene. Each siRNA specifically reduced the target mRNA and protein levels (Figure 4a,b). The number of viable cells, which was increased 1.4-fold by TNF-α stimulation, was substantially decreased by *CDK6* siRNA. However, no statistical changes were observed following treatment with *CDK4* siRNA (Figure 4c). *RB1* mRNA expression induced by TNF-α stimulation was markedly suppressed under both *CDK4/6* siRNA conditions, and the RB protein expression was downregulated under the same conditions (Figure 4a,b). In contrast, pRB expression was markedly decreased under *CDK6*, but not under *CDK4*, siRNA conditions (Figure 4b).

## 3. Discussion

In this study, we demonstrated that CDK6 is critical for kinase-dependent cell proliferation during the G1/S transition in cytokine-stimulated RASFs. A recent study has shown that CDK6, but not CDK4, expressed in RASFs and B cells, may contribute to the heritability of RA [26]. Our data translated genome-wide association study (GWAS) findings to the potential therapeutic value in terms of CDK6 expression in RASFs.

I3C has been reported to exhibit anti-inflammatory and antioxidant effects in a rat model of adjuvant-induced arthritis [27]. The dietary dose of I3C (1 μmole/g powdered chow) used in this study was the same as that in a previous study involving a lung tumor mouse model [28]. I3C considerably suppressed arthritic severity (Figure 1a,b), with no myelosuppressive adverse effects (Figure 1d), unlike that observed in CIA mice treated with palbociclib [19]. Additionally, there was no obvious influence on serum anti-CII IgG levels (Figure 1c).

The in vitro experiments indicated that I3C inhibited TNF-α-induced RASF proliferation (Figure 2a). Treatment with 200 μM I3C effectively suppressed CDK6 expression at both mRNA and protein levels (Figure 2b,c), which is consistent with a report on MCF7 cells [29]. I3C inhibited RB expression and phosphorylation to levels similar to those by palbociclib (Figure 2c). It has been reported that the antiproliferative effects of I3C induce G1 phase arrest and inhibit RB phosphorylation [30]. In TNF-α-stimulated RASFs, I3C reduced cell viability and substantially inhibited RB phosphorylation, but did not markedly suppress CDK6 expression, as previously reported in MCF7 cells [25]. Microarray analysis of prostate cancer cells (PC3) indicates that I3C influences the expression of many genes related to cell cycle, proliferation, and apoptosis [31,32]. It remains to be determined whether the observed inhibition of RASF proliferation and RB phosphorylation by I3C treatment was due to a modulatory effect of the suppression of CDK6 expression.

We demonstrated a direct role of CDK6 in the marked suppression of CIA in mice (Figure 3a). The histological score of the knee joints was also reduced, with no scores above two, which is defined as synovitis (Figure 3b). In other words, *Cdk6* deficiency resulted in the lack of hyperplasia and pannus formation in CIA. These results indicate that the progression of CIA synovial hyperplasia is dependent on CDK6, but not CDK4, at least in mice.

CDK6 is abundant in lymphocytes that mediate acquired immune responses [33]. *Cdk6*-deficient mice have been shown to exhibit delayed T-cell proliferation in response to mitogenic stimuli [11]. They may exhibit delayed acquired immune responses compared to WT mice. In the present study, *Cdk6*-deficient CIA mice showed a marked decrease in serum anti-CII IgG levels compared to WT mice (Figure 3c). Presumably, the decrease in serum anti-CII IgG levels was a consequence of T-cell dysfunction due to *Cdk6* deficiency. However, our results cannot exclude the influence of the immune system on the marked improvement in CIA induced by *Cdk6* deficiency.

Although complete deletion of CDK6 raises concerns about vulnerability to infection, the known decrease in all peripheral blood parameters in CIA mice treated with palbociclib [19] was not observed in *Cdk6*-deficient mice (Figure 3d). No significant reductions in neutrophil and other immune cell counts (CD115^+^ monocytes, CD4^+^ T cells, CD8^+^ T cells, and B220^+^ B cells) have been reported in non-immunized *Cdk6*-deficient mice [34]. Studies have also shown that a 15% reduction in red blood cell counts in non-immunized *Cdk6*-deficient mice did not result in any major physiological consequences [35]. In contrast, hematopoietic defects that cause embryonic lethality in *Cdk4/6* double-deficient mice are attributed to reduced numbers of hematopoietic progenitors and mature cells [11]. Based on the above, CDK4 expression might compensate for the maintenance of peripheral blood cell counts in *Cdk6*-deficient mice. Our findings suggest that selective inhibition of CDK6 expression, rather than the use of CDK4/6 dual inhibitors such as palbociclib, during RA treatment, may preclude the adverse effects of myelosuppression and offer potential therapeutic benefits in RA.

CDK4/6 generally share overlapping mechanisms through cell cycle progression. For non-overlapping mechanisms of CDK4/6, cell type-specific expression has been suggested to explain their differential requirements [36,37,38]. The present study further highlights the differential requirements of CDK4/6 in RASF proliferation. *CDK6* silencing was found to substantially reduce the number of TNF-α-induced viable cells (Figure 4c). CDK4 and CDK6 had comparable expression levels in the absence of TNF-α stimulation; TNF-α stimulation upregulated CDK6 expression but decreased CDK4 expression. Transcriptome analysis was used to examine the gene expression profiles of G1/S-phase-related factors, which undergo expression changes upon TNF-α stimulation in RASFs; only *CDK6* showed a substantially increased log_2_ ratio of more than 2 (Figure S1). In the presence of TNF-α, CDK6 expression was elevated by *CDK4* siRNA at both the mRNA and protein levels, whereas CDK4 expression remained constant with *CDK6* siRNA (Figure 4a,b). This finding suggests that the functional loss of CDK4 may be compensated for by CDK6, but not vice versa. In addition, the pRB level was not suppressed by *CDK4* siRNA. This observation indicates that CDK6, but not CDK4, is involved in G1 phase progression by regulating RB phosphorylation in TNF-α-induced RASF proliferation.

Current RA therapies target immune inflammation, and efforts to target synovial fibroblasts in the clinic are in their infancy. The efficacy of CDK inhibitors in suppressing RASF proliferation has been evaluated recently. A phase 1b trial of orally available seliciclib showed a trend toward decreased disease activity and no myelosuppression [39]. The novel CDK4/6 inhibitor TCK-276 has a significantly shorter half-life than conventional CDK4/6 inhibitors, and in the phase 1b trial, it reduced disease activity in the absence of myelosuppression [40]. Our study presents a safe, myelosuppression-avoiding treatment option that selectively targets a single molecule, CDK6, rather than both CDK4 and CDK6 and may lead to remission in patients with RA.

## 4. Materials and Methods

### 4.1. Mice

Male DBA/1J mice were purchased from Charles River Laboratories (Tokyo, Japan) and acclimatized for 1 week at our facility under conventional conditions at a temperature of 23 °C ± 1 °C, humidity of 55% ± 5%, and 12:12 h dark/light cycle. The mice had ad libitum access to food (CE-2; CLEA, Tokyo, Japan) and tap water. Mice (Cdk6tm1Bbd) on a C57BL/6 background [11] were gifted by Dr. M Barbacid; they were backcrossed with DBA/1J mice for ≥10 generations. Mouse littermates were used in the experiment.

### 4.2. Cell Culture

Anonymized, patient-derived RASFs were used in the experiments. Primary RASFs were isolated using an established method [8] and cultured (under 5% CO_2_ at 37 °C) in Dulbecco’s modified Eagle’s medium (DMEM; 043-30085; FUJIFILM Wako Pure Chemical, Tokyo, Japan) supplemented with 1% penicillin/streptomycin (Thermo Fisher Scientific, Waltham, MA, USA) and 10% heat-inactivated fetal bovine serum (FBS; Corning, One Riverfront Plaza Corning, NY, USA).

### 4.3. Reagents

For in vitro studies, TNF-α (T6674; Merck, Darmstadt, Germany) was dissolved in distilled water, while palbociclib (PZ0383; Merck) and I3C (I7256; Merck) were dissolved in dimethyl sulfoxide (DMSO) and diluted in culture medium. Mice were fed powdered chow (CE-2; CLEA) with or without 1 μmole of I3C per gram, starting from day 21 after the first immunization until the end of the experiment. The diet was replaced every 3 days.

### 4.4. siRNA

CDK4-targeted (SASI_Hs01_00122488), CDK6-targeted (SASI_Hs01_00048790), and MISSION universal negative control (SIC-001) siRNAs were obtained from Merck. Fifty percent of confluent cells cultured in DMEM containing 10% FBS were transfected with siRNA using Lipofectamine 2000 (Thermo Fisher Scientific) and Opti-MEM (Thermo Fisher Scientific). A total of 200 pmol of siRNAs were used in 6-well plates.

### 4.5. Quantitative RT-PCR

The cDNA was reverse transcribed from the total RNA using ReverTra Ace (TOYOBO, Osaka, Japan). Target genes were amplified using the ABI StepOne real-time PCR system (Thermo Fisher Scientific) with TB Green Premix Ex Taq (TaKaRa Bio, Shiga, Japan) and specific primers: *CDK4*, 5′-tgcagtcggtggtacctgagatg-3′ (forward) and 5′-gctcaccggattaccttcatcc-3′ (reverse) (product length, 144 bp); *CDK6*, 5′-gctgtctcactctagcaaccatcc-3′ (forward) and 5′-tcagagcattctgaagacagtagcc-3′ (reverse) (product length, 113 bp); *RB1*, 5′-ccagacccagaagccattga-3′ (forward) and 5′-ttcacaaagtgtatttagccggaga-3′ (reverse) (product length, 93 bp); glyceraldehyde-3-phosphate dehydrogenase (*GAPDH*), 5′-gcaccgtcaaggctgagaac-3′ (forward) and 5′-atggtggtgaagacgccagt-3′ (reverse) (product length, 141 bp). *GAPDH* was used for normalization. Each sample was analyzed in triplicate, and the relative mRNA expression level of the control without TNF-α stimulation was set to 1 using the 2^−ΔΔCt^ relative expression method [41].

### 4.6. Cell Viability Assay

The cells were treated with inhibitors (Figure 2a) or transfected with siRNAs (Figure 4c) and stimulated with PBS or TNF-α, according to the experimental conditions. The Cell Counting Kit-8 (CCK-8) (Dojindo Laboratories, Kumamoto, Japan) assay was performed, and the absorbance was measured at 450 nm using a Multiskan FC microplate photometer (Thermo Fisher Scientific). Relative cell viability is expressed as 1, using siRNA-transfected cells without TNF-α stimulation as the control.

### 4.7. Western Blotting

The cells were lysed in ice-cold RIPA (radio-immunoprecipitation assay) buffer (20 mM HEPES pH 7.5, 150 mM NaCl, 1% NP-40, 0.5% sodium deoxycholate) containing protease inhibitors (ATTO, Tokyo, Japan) and phosphatase inhibitors (ATTO). Proteins (10 μg) were electrophoresed on 12.5% e-PAGEL HR (ATTO) using EzRun MOPS buffer (ATTO). After transferring the proteins onto polyvinylidene fluoride membranes (Bio-Rad, Hercules, CA, USA), the membranes were evaluated as previously described [8,9], using anti-CDK4 (C8218; Merck; 1:1000), anti-CDK6 (sc-390493; Santa Cruz, Dallas, TX, USA; 1:1000), anti-RB (9309; Cell Signaling Technology, Danvers, MA, USA; 1:1000), anti-pRB S807-811 (9308; Cell Signaling Technology; 1:1000), or anti-α-TUB antibodies (HRP-66031; Proteintech, Rosemont, IL, USA; 1:15,000), followed by the corresponding horseradish peroxidase (HRP)-conjugated secondary antibody (DAKO, Carpinteria CA, USA). The signals were visualized using Immobilon Western Chemiluminescent HRP substrate (Merck) and detected with LAS-4000 (GE Healthcare, Chicago, IL, USA).

### 4.8. Induction and Assessment of CIA

Male mice were randomly allocated to different groups. Mice aged 9–10 weeks were immunized with 100 μg bovine type II collagen (K42; Collagen Research Center, Tokyo, Japan) at the base of their tails. Emulsions were prepared using Freund’s complete adjuvant (263810; BD Biosciences, Franklin Lakes, NJ, USA) the first time and Freund’s incomplete adjuvant (263910; BD Biosciences) the second time. Subsequently, the arthritis scores were assessed three times a week until the incidence of arthritis in control mice reached 100%. Each paw was scored semi-quantitatively from 0 to 4, with a maximum limb score of 16 per mouse [8,9]. All mice were sacrificed via decapitation under isoflurane anesthesia (FUJIFILM Wako Pure Chemical), and a blinding strategy was applied by concealing labels when collecting their blood samples (serum and peripheral blood) and knee joints.

### 4.9. Histological Analysis

Knee joints were post-fixed in 10% formalin, decalcified in 10% ethylenediaminetetraacetic acid (EDTA), and embedded in paraffin. Sagittal sections (4 μm) were stained with hematoxylin and eosin (HE) and evaluated according to the pathological features of synovial surface cell proliferation, mononuclear cell infiltration, and pannus formation, including synovial hyperplasia. The slides were scored from 0 (no inflammation) to 4 (pannus formation and cartilage erosion) [42], as previously described [8,9]. In particular, a sectional image with two to three layers of synovial lining cells and scattered mononuclear infiltrates was assigned a score of 2 (definite arthritis). Images were captured using a BZ-X700 microscope (Keyence, Osaka, Japan) equipped with a 10× objective lens.

### 4.10. Mouse Anti-Bovine Type II Collagen IgG Antibody Assay

Serum samples were collected and measured according to the protocol of the Mouse Anti-Type II Collagen IgG Assay ELISA kit (500410; Cayman Chemical Company, Ann Arbor, MI, USA).

### 4.11. Hematological Examination

Peripheral blood samples were collected in EDTA 2 K-coated MiniCollect tubes (Greiner Bio-One, Kremsmunster, Austria). Blood cells were enumerated at the Sanritsu Zelkova Veterinary Laboratory (Tokyo, Japan). Neutrophils and lymphocytes were automatically classified based on leucocyte morphology (%).

### 4.12. Ethical Statement

Synovial tissues from human joint replacement surgeries were collected following a protocol (No. 1315), approved by the Ethics Review Committee of the St. Marianna University School of Medicine, and complied with the tenets of the Declaration of Helsinki. Written informed consent was obtained from all patients. RASFs were used in independent experiments with different cells derived from three patients.

Animal experiments were conducted in accordance with animal experimental protocols (No. TG230324-6C) and approved by the Animal Experiment Committee of the St. Marianna University School of Medicine. The experiments also complied with the ARRIVE guidelines.

### 4.13. Statistical Analysis

The sample size is listed in the figure legend for each experiment. For in vivo stud-ies, power and sample size calculations of the primary outcome (the mean arthritis score on the last day of observations in CIA mice) were performed using JMP Pro 17 software based on the mean difference between independent groups, with a power (1-β error probability) of 83.60%, effect size (d) of 2.5, α error probability of 5%, and a two-tailed test. The minimum number of mice estimated for each group was four.

All experimental analyses were performed using GraphPad Prism 10. Fisher’s exact test was used to compare the incidence of arthritis (Figure 1a and Figure 3a). The Mann–Whitney U test was used to compare the arthritis (Figure 1a and Figure 3a) and histological scores (Figure 1b and Figure 3b). Welch’s *t*-test was used for comparisons between groups (Figure 1c,d and Figure 3c,d), whereas one-way analysis of variance, followed by Dunnett’s multiple comparison test, was used for comparisons against TNF-α -stimulated RASFs for three independent experiments (Figure 2a,b and Figure 4a,c). No inclusion or exclusion criteria were used for experimental data. Significance was determined using two-tailed *p*-values < 0.05.

## 5. Conclusions

The inhibition of CDK6 expression in RASFs, without functional compensation from CDK4, contributes to the suppression of RB phosphorylation, which is crucial for the progression of TNF-α-induced cell proliferation. From the results obtained using pharmacological blockades and genetically deficient mice, CDK6 is involved in the prevention of synovial hyperplasia, which is important for pannus formation, causing bone and cartilage damage. This approach to modulate CDK6 expression without complete loss of CDK6 or suppression of CDK4 expression may have an ameliorative effect on RASF proliferation in the joints of patients with RA without causing excessive immunosuppression.

## Data Availability

The data that support the findings of this study are included in the article/Supplementary Material. Further inquiries can be directed to the corresponding author.

## Supplementary Materials

The following supporting information can be downloaded at: https://www.mdpi.com/article/10.3390/ijms26031151/s1.
