# Supplementary material for: CDK6-Dependent, CDK4-Independent Synovial Hyperplasia in Arthritic Mice and Tumor Necrosis Factor-α-Induced Proliferation of Synovial Fibroblasts
Source: Int J Mol Sci. 2025 Jan 28;26(3):1151. doi: 10.3390/ijms26031151 (PMC11817658; doi:10.3390/ijms26031151)
Supplement: Supplementary file 1 [file ijms-26-01151-s001.zip › ijms-3410943-supplementary.pdf]

Supplementary material

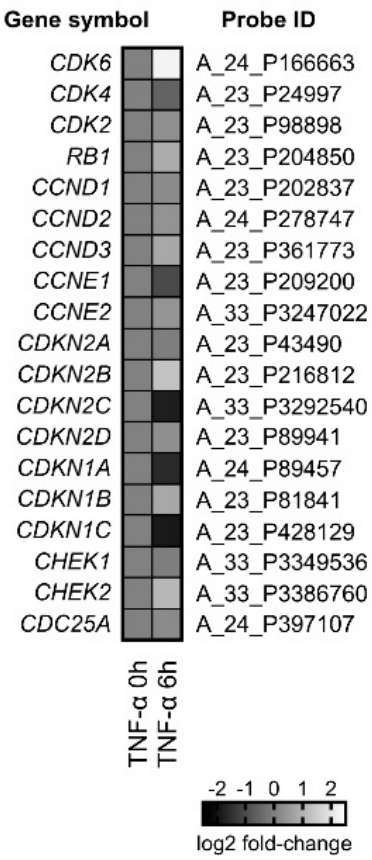

Figure S1. Gene expression profile of G1/S-phase-related factors. Cells were collected using ISOGEN (Nippon Gene, Tokyo, Japan) at 0 or 6 h after TNF-α stimulation, and total RNA was extracted using the RNeasy Mini Kit (QIAGEN, Hilden, Germany). DNA array analysis was performed using Agilent SurePrint G3 Human GE 8 × 60 K v2 Microarray (TaKaRa Bio, Shiga, Japan). The heatmap displays the log_2 expression ratio of genes associated with the G1/S phase following TNF-α (10 ng/mL) stimulation. Gene symbol (name of the gene encoded by the probe) and probe ID are from the microarray
